# 7-Ketocholesterol and cholestane-triol increase expression of SMO and LXRα signaling pathways in a human breast cancer cell line

**DOI:** 10.1016/j.bbrep.2018.12.008

**Published:** 2018-12-31

**Authors:** Debora Levy, Thatiana Correa de Melo, Beatriz A. Oliveira, Jessica L. Paz, Fabio A. de Freitas, Cadiele O. Reichert, Alessandro Rodrigues, Sergio P. Bydlowski

**Affiliations:** a Laboratory of Genetics and Molecular Hematology (LIM31), Department of Hematology, Hospital das Clínicas HCFMUSP, Faculdade de Medicina, Universidade de Sao Paulo, São Paulo, SP, Brazil; b Departamento de Química, Universidade Federal de Sao Paulo, Diadema, SP, Brazil; c Instituto Nacional de Ciencia e Tecnologia em Medicina Regenerativa (INCT-Regenera), CNPq, Brazil

**Keywords:** Oxysterol, Apoptosis, Cancer, ABC transporters, Sonic hedgehog, LXRα

## Abstract

Oxysterols are 27-carbon oxidation products of cholesterol metabolism. Oxysterols possess several biological actions, including the promotion of cell death. Here, we examined the ability of 7-ketocholesterol (7-KC), cholestane-3β-5α-6β-triol (triol), and a mixture of 5α-cholestane-3β,6β-diol and 5α-cholestane-3β,6α-diol (diol) to promote cell death in a human breast cancer cell line (MDA-MB-231). We determined cell viability, after 24-h incubation with oxysterols. These oxysterols promoted apoptosis. At least part of the observed effects promoted by 7-KC and triol arose from an increase in the expression of the sonic hedgehog pathway mediator, smoothened. However, this increased expression was apparently independent of sonic hedgehog expression, which did not change. Moreover, these oxysterols led to increased expression of LXRα, which is involved in cellular cholesterol efflux, and the ATP-binding cassette transporters, ABCA1 and ABCG1. Diols did not affect these pathways. These results suggested that the sonic hedgehog and LXRα pathways might be involved in the apoptotic process promoted by 7-KC and triol.

## Highlights

• Oxysterols (7-KC, triol, and diol) promoted apoptosis in a human breast cell line.
• Sonic hedgehog expression did not change as effect of 7-KC and triol.
• Smoothened expression increased as effect of 7-KC and triol.
• An increased expression of LXRα, ABCA1, and ABCG1 was also observed.
• SMO and LXRα may be involved in the apoptotic process promoted by 7-KC and triol.

## 1 Introduction

Cholesterol is an essential component of cell membrane and also a precursor of steroid hormones and bile acids [1]. Oxysterols are a large family of 27-carbon oxidized derivatives of cholesterol and similarly, they are made of a steroid backbone and a side chain [2]. Endogenous oxysterols are formed either by auto-oxidation or by enzyme-mediated mechanisms [3], [4], [5]. Oxidation promotes the addition of hydroxyl, keto, hyperoxy, carbonyl, or epoxy groups to the cholesterol backbone, mostly at the C4–7 positions or at the C24, 25, and 27 positions of the lateral chain [6]. This process generates a large class of different oxysterols.

Bioactive lipids are endogenous lipid mediators that have functional actions. Several elements point to oxysterols as bioactive lipids since they are involved in a plethora of physiological and pathophysiological processes. In fact, several oxysterols are biologically active as regulatory molecules. For example, they regulate sterol and lipid metabolism, modulate signaling pathways, and influence cell proliferation and differentiation [6]. Consequently, oxysterols participate in a variety of pathophysiological processes, including atherosclerosis and cancer [7], [8], [9], [10], [11], [12]. Moreover, some oxysterols (e.g. 4β-hydroxycholesterol or 7α-hydroxycholestenone) are used as biomarkers of specific pathologies [12].

One particularly interesting effect of several oxysterols is the promotion of cell death. This effect has been observed in a number of cell lines [11], [13], [14], [15], [16], and it occurs through several mechanisms, including gene expression regulation [4], apoptosis, and autophagy induction [13], [17], [18]. Many cell types are sensitive to the cytostatic and cytotoxic effects of oxysterols, including several tumor cell lines [6], [19], [20], [21], [22].

These properties led us to investigate the potential use of oxysterols as chemotherapeutic agents in cancer [23]. Our results showed that 7-ketocholesterol (7-KC), a well-known oxysterol, had cytostatic and cytotoxic effects on melanoma, in vitro and in vivo. Therefore, we hypothesized that 7-KC cytotoxicity could be applied in cancer therapeutics. However, it has also been shown that the cytotoxic effect of oxysterols on cells varies according to both the type of oxysterol and the specific cell line [24]. This is not surprising considering the large number of molecules within this family and their complex metabolism [12]. Another reason is the large number of molecular targets identified for these bioactive lipids. From a functional point of view, the proteins that bind the oxysterols can be classified into receptors (nuclear and GPCRs) and regulatory or transport proteins [12].

Liver X receptors (LXR) were the first nuclear receptors described for oxysterols. LXR form a heterodimer with the retinoic X receptor (RXR). Following activation by oxysterols (LXR) or by retinoic acid (RXR), the heterodimer recruits co-activators proteins and can initiate transcriptional activity [12]. The genes targeted by the heterodimer LXR/RXR are mainly those involved in the reverse cholesterol transport, such as ATP-binding cassette transporters [25]. Importantly, not all the oxysterols behave as full LXR agonists; several of them actually behave as antagonists [12]. Among the cell membrane receptors known to bind oxysterols, the GPCR smoothened (SMO) was found to be activated by some oxysterols [26], [27].

Previously, we described the cytotoxic effect of 7-ketocholesterol, cholestane-triol, and cholestane-diol on several cell lines [24]. These oxysterols inhibited the S phase and stimulated the G0/G1 or G2/M phases. They also promoted apoptosis, as determined with Annexin V and propidium iodide assays. Here, we explore more deeply the apoptotic effect of these cholesterol oxides further by assessing their ability to affect Smoothened (SMO) and Sonic Hedgehog (SHh) expression as well as the expression of LXRα and ABCA1 and ABCG1 transporters, which could also be involved in the apoptotic process.

## 2 Materials and methods

### 2.1 Oxysterol solution preparation

7-ketocholesterol, cholestane-3β-5α-6β-triol, and a mixture of 5α-cholestane-3β,6β-diol and 5α-cholestane-3β,6α-diol were synthesized as described [24, Supplementary materials]. All other reagents and solvents were purchased from Sigma-Aldrich (MO, USA). All commercially available chemicals were used without purification. Stock solutions for each compound were prepared at a concentration of 1000 μM in absolute ethanol.

### 2.2 Cell culture and oxysterol treatment

A human mammary gland/breast cell line, derived from a metastatic site, (MDA-MB-231; ATCC HTB-26) was used in this study. Unless otherwise specified, reagents for culture procedures were purchased from Sigma. Culture contamination by mycoplasma was routinely tested with DAPI staining.

After thawing [28], cells were grown in Dulbecco's Modified Eagle's Medium (DMEM) supplemented with 10% fetal bovine serum (FBS, Gibco, Karlsruhe, Germany), 100 U/mL penicillin, and 100 mg/mL streptomycin for 48 h, then plated at a density of 5 × 10^3^ cells/cm^3^ in 96-well black flat bottom polystyrene microplates (Corning, MA). Cells were maintained at 37 °C under a humidified atmosphere with 5% CO_2_ [24].

Various concentrations of oxysterols (0–100 μM, 100 µL final volume) were added to cells and incubated for 24 h. Then, we tested cell viability and performed indirect immunofluorescence detection of SMO, SHh, LXRα, ABCA1, and ABCG1.

### 2.3 Cell viability assay

After 24-h treatment with oxysterols, cells were incubated with 0.1 μg/mL Hoechst 33342 (H1399- Molecular Probes, OR, USA) and 0.5 µL propidium iodide (PI) (P3566- Molecular Probes) for 15 min. An ImageXpress Micro high-content screening system (Molecular Devices, CA, USA) was used to determine the number of live and dead cells. Nine sites per well and three wells per treatment were evaluated. Cell Scoring MetaXpress software was used to analyze the number of cells and cell viability. For IC_50_ calculations, we evaluated survival data with a variable slope curve-fitting application provided in GraphPad Prism (GraphPad Software, CA, USA).

### 2.4 Indirect immunofluorescence detection of smoothened (SMO), sonic hedgehog (SHh), liver X receptor (LXRα), and ATP-binding cassette transporters (ABCA1 and ABCG1)

For all indirect immunofluorescence experiments, cells were plated at a density of 1.5 × 10^3^ cells/cm^2^ in triplicate in 96-well black flat-bottom polystyrene microplates (3603- Corning). Cells were treated with different oxysterols for 24 h, and fixed in 4% paraformaldehyde (P-6148 Sigma Aldrich) for 2 h at 4 °C. After washing with DPBS, cells were permeabilized with 0.1% Triton X-100 solution (93443 Sigma Aldrich) at 4 °C for 15 min, followed by blocking with 5% BSA (A9418 Sigma Aldrich) for 40 min at room temperature. Cells were incubated overnight with the following antibodies: anti-SMO (1:100 dilution; NBP2–24543-Novus Biologicals, CO, USA); anti-SHh (1:400 dilution; ab53281- Abcam, Cambridge, UK); anti-LXRα (1:50 dilution; ab41902 Abcam); anti-ABCA1 (1:50 dilution; NB100–2068- Novus Biologicals); and anti-ABCG1 (1:50 dilution; H9619-M03- Abnova, Taipei, Taiwan). After incubating with anti-SMO and anti-SHh antibodies, cells were incubated for 1 h more with the anti-rabbit secondary antibody, AlexaFluor 488 (1:1000 dilution; A-11008- Molecular Probes, OR, USA). Cells incubated with antibodies against LXRα, ABCA1, and ABCG1 were incubated for 1 h with the anti-mouse secondary antibody, R-phycoerythrin (1:1000 dilution; P852- Molecular Probes). The fluorescence intensities of SMO, SHh, LXRα, ABCA1, and ABCG1 labeling were determined with ImageXpress. Nine sites per well and 3 wells per treatment were evaluated. Data were analized with the cell scoring MetaXpress software.

### 2.5 Statistical analysis

Data are expressed as the mean ± SD of at least three independent experiments. Means were compared with the Mann-Whitney *U* test provided in GraphPad Prism (GraphPad Software, CA). P-values ≤ 0.05 were considered significant.

## 3 Results and discussion

Bioactive lipids are known to regulate several cellular processes [29], [30], [31], [32], including cell growth, proliferation, differentiation, and death [33], [34]. Among the bioactive lipids, oxysterols are potent, biologically active molecules, involved in several cell functions, including inhibiting cell proliferation and promoting cell death [35], [36]. The most studied oxysterols, at least in terms of toxicity, are 25-hydroxycholesterol, 7β-hydroxycholesterol, and 7-ketocholesterol. The cytotoxic effects of these oxysterols have been demonstrated in several cell lines [16], [22], [35], [37], [38], [39], [40], [41], [42].

Here, as expected, 7-KC, triol, and diol reduced the number of cells. As described previously, apoptosis was involved as a cause of cell death [24]. We explored the mechanisms of apoptosis promoted by 7-KC, triol, and diol by evaluating the effects of subtoxic doses (30 μM) on the sonic hedgehog (SHh) pathway and liver X receptor alpha (LXRα). SHh can cause different effects on cells at different concentrations. The SHh pathway is activated when SHh binds to its receptor, the transmembrane protein, Patched (PTCH) [43]. PTCH proteins prevent downstream signaling by attenuating Smoothened (SMO) activity [44]. However, when SHh binds to PTCH, it removes the repression of SMO, which then activates a signal transduction pathway in the cytoplasm [45]. Recently, it was shown that oxysterols could allosterically activate SMO by binding to its extracellular cysteine-rich domain [46].

**Fig. 1:**
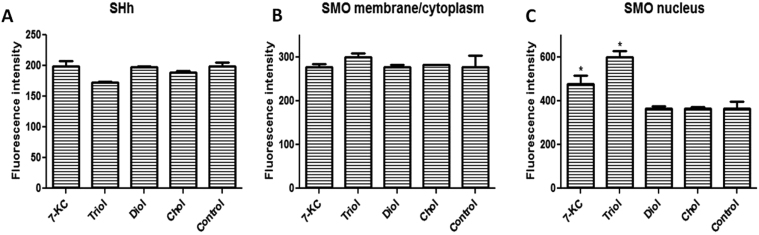
Immunofluorescence detection of sonic hedgehog (SHh) and smoothened (SMO) expression in MDA-MB-231 cell line after 24 h incubation with 30 µM oxysterols. **A:** SHh expression; **B:** SMO expression in the membrane/cytoplasm;**C:** SMO expression in the nucleus. The intensity of fluorescence was quantified with MetaXpress software. Abbreviations: 7KC: 7-ketocholesterol; triol: cholestan-3α-5β-6α-triol; diol: 5α-cholestane-3β,6β-diol/5α-cholestane-3β,6α-diol. Cholesterol was used as control. Data are presented as the mean ± SEM from three independent experiments performed in triplicate. *p < 0.005 compared to control.

Here, we evaluated SHh with immunofluorescence. None of the oxysterols or cholesterol (as control) changed SHh protein expression (Fig. 1A). The effect of oxysterols on SMO was evaluated by assessing fluorescence intensity in the membrane/cytoplasm and in the nucleus. Cells expressed SMO protein. Neither oxysterols nor cholesterol changed SMO expression in the membrane/cytoplasm (Fig. 1B). On the other hand, SMO expression in the nucleus increased lines after treatment with 7-KC and triol (Fig. 1C). Cholesterol had no effect on nuclear SMO levels. Therefore, these oxysterols did not appear to act on SMO by changing SHh expression, but a possible direct action on SMO should be considered.

LXRs are nuclear receptors with important roles in the transcriptional control of lipid metabolism. They were initially described as orphan receptors, but later, oxysterols were identified as their natural ligands. Activated LXRs form heterodimeric complexes with retinoic acid receptors (RXRs) [47]. LXRs exert important effects, including control of transcription factors and gene regulation. The genes targeted by LXR/RXR are mainly involved in cholesterol efflux from cells (reverse cholesterol transport) through the ATP-binding cassette transporters, ABCA1, ABCG5, ABCG8, and ABCG1 [12], [48]. It is well known that cholesterol metabolism is dysregulated in different malignant cells. LXRs have been described as having anticancer properties. They can regulate tumor growth in various cancer cell lines [49], [50], [51]. In the past few years, anti-proliferative effects of synthetic and natural LXR agonists have been observed in various types of human cancer, in vitro and in vivo: blastic plasmacytoid dendritic cell neoplasm [49], prostate cancer cells [52], melanoma [53], colon cancer cells [54], acute lymphoblastic leukemia [55], human lung cancer [56]. Therefore, LXR agonists have been considered as potential anti-cancer agents. It has been proposed that activation of LXR deprives cancer cell membranes of lipids essential for their growth, inhibiting cell proliferation, by stimulating cholesterol efflux via upregulation of ABCA1 and ABCG1 [48], [49]. However, whether these effects are related only to cholesterol efflux has not yet been elucidated.

**Fig. 2:**
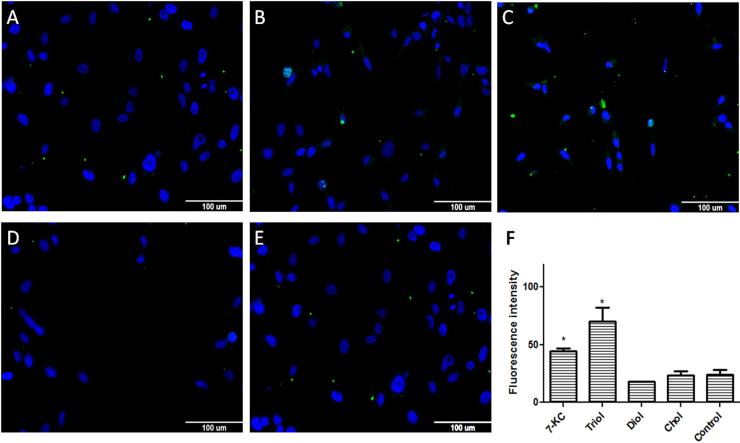
Immunofluorescence detection of LXRα expression in the MDA-MB-231 cell line after 24 h incubation with 30 µM of oxysterols. The intensity of fluorescence was quantified with MetaXpress software. Representative images of immunofluorescence after incubations with **A:** 7-ketocholesterol (7KC); **B**: cholestan-3α-5β-6α-triol (triol); **C**: 5α-cholestane-3β,6β-diol/5α-cholestane-3β,6α-diol (diol); or **D:** cholesterol (control); **E:** quantification of LXRα expression. Data are presented as the mean ± SEM from three independent experiments performed in triplicate. *p < 0.005 compared to control.

**Fig. 3:**
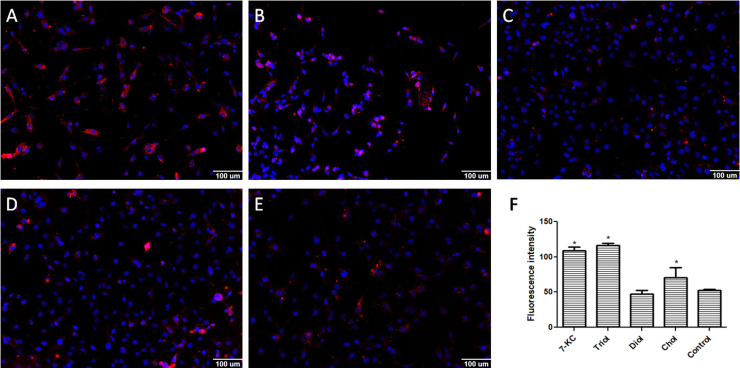
Immunofluorescence detection of ABCA1 expression in the MDA-MB-231 cell line after 24 h incubation with 30 µM of oxysterols. The intensity of fluorescence was quantified with MetaXpress software. Representative images of immunofluorescence after incubations with **A:** 7-ketocholesterol (7KC); **B**: cholestan-3α-5β-6α-triol (triol); **C**: 5α-cholestane-3β,6β-diol/5α-cholestane-3β,6α-diol (diol); or **D:** cholesterol (control); **E:** quantification of ABCA1 expression. Data are presented as the mean ± SEM from three independent experiments performed in triplicate. *p < 0.005 compared to control.

**Fig. 4:**
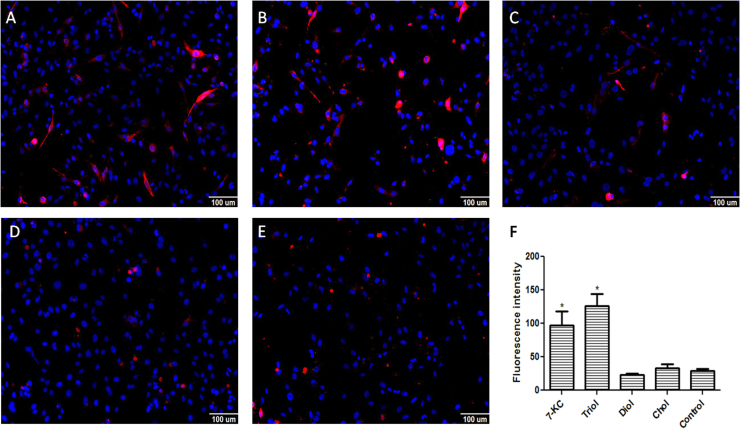
Immunofluorescence detection of ABCG1 expression in the MDA-MB-231 cell line after 24 h incubation with 30 µM of oxysterols. The intensity of fluorescence was quantified with MetaXpress software. Representative images of immunofluorescence after incubations with **A:** 7-ketocholesterol (7KC); **B**: cholestan-3α-5β-6α-triol (triol); **C**: 5α-cholestane-3β,6β-diol/5α-cholestane-3β,6α-diol (diol); or **D:** cholesterol (control); **E:** quantification of ABCG1 expression. Data are presented as the mean ± SEM from three independent experiments performed in triplicate. *p < 0.005 compared to control.

We evaluated the effect of 7-KC, triol, diol, and cholesterol (30 μM) on LXRα, ABCA1, and ABCG1 expression. LXRα fluorescence intensity increased when cells were treated with 7-KC and triol, but not with diol or cholesterol (Fig. 2). Based on these results, we tested ABCA1 and ABCG1 expression (Fig. 3, Fig. 4, respectively). Again, cells treated with 7-KC and triol showed elevated expression of ABCA1 and ABCG1 proteins, but no change was observed with diol treatment. Cholesterol caused small increases in ABCA1 expression, as expected, but no effect was observed on ABCG1 expression. Therefore, we hypothesized that 7-KC and triol might increase ABCA1 and ABCG1 expression levels by stimulating LXRα. This can, at least in part, contribute for the observed effects on cell proliferation and death. Besides, LXRα could also contribute to the process of apoptosis, as recently shown. In addition to the inhibition of cancer cell survival related to cholesterol deprivation, LXRs also control the expression of genes involved in many other processes [12]. Treatment with LXR agonists was described to be responsible for inducing intrinsic apoptotic cell death [49]. Increased apoptotic rates have been promoted by several mechanisms: up-regulation of the pro-apoptotic gene BAX and reduction of the anti-apoptotic gene BCL-2 expression [48], downregulation of AKT survival signaling [52], [56], caspase-3 pathway [53], caspase-1 dependent cell death induction [54], up-regulation of SOCS3 pathway [55]. It has also been suggested that ABCA1 and ABCG1 are required for the apoptotic clearance process, including appropriate phagocytosis of apoptotic cells [50], [51].

In conclusion, cytotoxic 7-ketocholesterol and cholestane-3β-5α-6β-triol could act on SMO and LXRα pathways to promote cell death. In view of these findings, their potential pharmacological utility merit further investigation.

## CRediT authorship contribution statement

**Debora Levy:** Conceptualization, Methodology, Investigation, Data curation, Formal analysis, Writing - original draft, Writing - review & editing. **Thatiana Correa de Melo:** Investigation, Data curation, Writing - review & editing. **Beatriz A. Oliveira:** Investigation, Data curation, Writing - review & editing. **Jessica L. Paz:** Investigation, Data curation, Writing - review & editing. **Fabio A. de Freitas:** Formal analysis, Writing - review & editing. **Cadiele O. Reichert:** Formal analysis, Writing - review & editing. **Alessandro Rodrigues:** Formal analysis, Writing - original draft, Writing - review & editing. **Sergio P. Bydlowski:** Conceptualization, Methodology, Formal analysis, Writing - original draft, Writing - review & editing, Supervision.
