# Supplementary material for: 7-Ketocholesterol and cholestane-triol increase expression of SMO and LXRα signaling pathways in a human breast cancer cell line
Source: Biochem Biophys Rep. 2018 Dec 31;19:100604. doi: 10.1016/j.bbrep.2018.12.008 (PMC6709374; doi:10.1016/j.bbrep.2018.12.008)
Supplement: Supplementary file 1 — Supplementary material [file mmc1.docx]

Hereby the authors declare no potential conflicts of interest regarding to the manuscript “7-Ketocholesterol and Cholestane-triol increase expression of SMO and LXRα Signaling Pathways in a Human Breast Cancer Cell Line”, by Debora Levy, Tathiana Correa de Melo, Beatriz A. Oliveira, Jessica L. Paz, Fabio A. de Freitas, Cadiele O. Reichert, Alessandro Rodrigues, and Sergio P. Bydlowski.
